# The Beta-2-Adrenoreceptor Agonists, Formoterol and Indacaterol, but Not Salbutamol, Effectively Suppress the Reactivity of Human Neutrophils *In Vitro*

**DOI:** 10.1155/2014/105420

**Published:** 2014-03-06

**Authors:** Ronald Anderson, Annette J. Theron, Helen C. Steel, Chrisna Durandt, Gregory R. Tintinger, Charles Feldman

**Affiliations:** ^1^Medical Research Council Unit for Inflammation and Immunity, Department of Immunology, Faculty of Health Sciences, University of Pretoria, P.O. Box 2034, Pretoria 0001, South Africa; ^2^Tshwane Academic Division of the National Health Laboratory Service, Pretoria 0001, South Africa; ^3^Division of Pulmonology, Department of Internal Medicine, Faculty of Health Sciences, University of the Witwatersrand and Charlotte Maxeke Johannesburg Academic Hospital, Johannesburg 2193, South Africa

## Abstract

The clinical relevance of the anti-inflammatory properties of beta-2 agonists remains contentious possibly due to differences in their molecular structures and agonist activities. The current study has compared the effects of 3 different categories of **β**2-agonists, namely, salbutamol (short-acting), formoterol (long-acting) and indacaterol (ultra-long-acting), at concentrations of 1–1000 nM, with human blood neutrophils *in vitro*. Neutrophils were activated with either N-formyl-L-methionyl-L-leucyl-L-phenylalanine (fMLP, 1 µM) or platelet-activating factor (PAF, 200 nM) in the absence and presence of the **β**2-agonists followed by measurement of the generation of reactive oxygen species and leukotriene B4, release of elastase, and expression of the **β**2-integrin, CR3, using a combination of chemiluminescence, ELISA, colorimetric, and flow cytometric procedures respectively. These were correlated with alterations in the concentrations of intracellular cyclic-AMP and cytosolic Ca^2+^. At the concentrations tested, formoterol and indacaterol caused equivalent, significant (*P* < 0.05 at 1–10 nM) dose-related inhibition of all of the pro-inflammatory activities tested, while salbutamol was much less effective (*P* < 0.05 at 100 nM and higher). Suppression of neutrophil reactivity was accompanied by elevations in intracellular cAMP and accelerated clearance of Ca^2+^ from the cytosol of activated neutrophils. These findings demonstrate that **β**2-agonists vary with respect to their suppressive effects on activated neutrophils.

## 1. Introduction

Beta-2-adrenoreceptor (*β*2ARs) agonists (*β*2-agonists) are used primarily as bronchodilators in the therapy of obstructive airways disorders, especially bronchial asthma and chronic obstructive pulmonary disease (COPD) [1, 2]. These agents also possess secondary anti-inflammatory properties, affecting cells of both the innate and adaptive immune systems and their inflammatory mediators* in vitro*, as well as in some animal models of experimental airway inflammation [3–5]. As with bronchodilatory activity, anti-inflammatory activity is achieved via sequential *β*2AR-mediated activation of adenylyl cyclase, elevated intracellular concentrations of 3′-5′-cyclic adenosine monophosphate (cAMP), and activation of cAMP-dependent protein kinase (PKA) [3–5]. While the therapeutic efficacy of the bronchodilatory actions of *β*2-agonists is undisputed, the clinical relevance of the anti-inflammatory activities of these agents remains uncertain [3, 5]. This contention is underscored by data from recently reported large, well-controlled clinical trials demonstrating lack of efficacy of aerosolized or intravenous salbutamol in the therapy of acute lung injury [6, 7]. Several mechanisms may underpin the apparent lack of anti-inflammatory potency of *β*2-agonists in the clinical setting. These include (i) lower numbers of *β*2-ARs on immune and inflammatory cells relative to airway smooth muscle cells, rendering them more susceptible to receptor desensitization [8], and (ii) possible variability in anti-inflammatory potency between different types of *β*2-agonists resulting from differences in molecular structure.

In the current study, we have compared the anti-inflammatory activities of two commonly used representatives of the short- and long-acting categories of *β*2-agonists (SABA, LABA), namely, salbutamol and formoterol, respectively, with those of the recently introduced ultralong-acting (ultra LABA), indacaterol, used for maintenance therapy of airflow limitation in patients with COPD [9, 10]. The study is focused on the effects of these agents on the proinflammatory activities of the human neutrophil, a cell type which not only expresses relatively high numbers of *β*2ARs in comparison with other types of immune and inflammatory cells [5, 11], but which is also intimately involved in the immunopathogenesis of COPD [12, 13]. In addition, while several recent studies have addressed the anti-inflammatory interactions of indacaterol with human lung fibroblasts [14] and mast cells [15], the effects of this agent on human neutrophils have not been described.

## 2. Materials and Methods

### 2.1. Chemicals and Reagents

Formoterol fumarate dehydrate and salbutamol hydrochloride were purchased from Sigma-Aldrich (Pty) Ltd. (Johannesburg, South Africa), while indacaterol maleate was provided by Novartis International AG, Basel, Switzerland. All 3 agents were dissolved to a stock concentration of 10 mM in dimethylsulfoxide (DMSO) and used at final concentrations of 1, 10, and 100 nM in most of the assays described below and at 1–1000 nM in others. The local concentration of formoterol following inhalation of 24 *μ*g of this agent has been estimated to be approximately 11 nM [16]. The final concentration of DMSO in each assay was 0.1% and appropriate solvent controls were included with each experimental system. Unless indicated, all other chemicals and reagents were purchased from Sigma-Aldrich.

Neutrophils were activated with the chemoattractant, N-formyl-L-methionyl-L-leucyl-L-phenylalanine (fMLP, 1 *μ*M final) either alone or in combination with cytochalasin B (CB, 1 *μ*M final) in assays of oxygen consumption and degranulation. An additional chemoattractant, platelet-activating factor (PAF, 200 nM final) was used in a more limited series of experiments.

### 2.2. Preparation of Neutrophils

The study was approved by the Faculty of Health Sciences Research Ethics Committee of the University of Pretoria, Pretoria, South Africa, and prior informed consent was obtained from all blood donors. Neutrophils were isolated from heparinized venous blood (5 units of preservative-free heparin per mL of blood) from nonsmoking healthy adult volunteers. Each subject completed a detailed health questionnaire and underwent a health check (including measurement of blood pressure) by an experienced, qualified nursing sister prior to venepuncture. Neutrophils were separated from mononuclear leucocytes by centrifugation on Histopaque-1077 (Sigma Diagnostics, St. Louis, MO, USA) cushions at 400 ×g for 25 min at room temperature. The resultant pellets were suspended in PBS (0.15 M, pH 7.4) and sedimented with 3% gelatin to remove most of the erythrocytes. Following centrifugation (280 ×g at 10°C for 10 min), residual erythrocytes were removed by selective lysis with 0.83% ammonium chloride at 4°C for 10 min. The neutrophils, which were routinely of high purity (>90%) and viability (>95%), were resuspended to 1 × 10^7^ cells mL^−1^ in PBS and held on ice until used.

### 2.3. Generation of Reactive Oxygen Species (ROS)

This was measured using a luminol (5-amino-2,3-dihydro-1,4-phthalazinedione)-enhanced chemiluminescence (LECL) procedure. Briefly, neutrophils (10^6^ cells) were preincubated for 10 min at 37°C in 900 *μ*L Hanks' balanced salt solution (HBSS, indicator-free; Highveld Biological (Pty) Ltd., Johannesburg, South Africa) containing luminol at a final concentration of 0.1 mM. Following preincubation, the test *β*2-agonists (1–1000 nM) or solvent control were added to the cells, followed after 20 s by fMLP (1 *μ*M), and LECL responses recorded using a Lumac Biocounter (model 2010; Lumac Systems Inc., Titusville, FL, USA). The final volume in each tube was 1 mL, and the results, which are expressed in relative light units (rlu), are the peak values for fMLP-activated systems that were reached 40–50 s after addition of stimulant.

### 2.4. Oxygen Consumption

This was measured using a 3-channel oxygen electrode (Model DW1, Hansatech Ltd., King's Lynn, Norfolk, UK). Neutrophils (2 × 10^6^) were incubated for 10 min at 37°C in HBSS after which the test *β*2-agonists, at a fixed, final concentration of 100 nM, were added to the cells, followed 20 s later by fMLP/CB (1 *μ*M : 1 *μ*M). PO_2_ was monitored over an 8 min period and O_2_ consumption calculated over the period when utilization was linear (1-2 min), which was approximately 1 min, and the results are expressed as nanomoles (nmols) O_2_ consumed min^−1^ by 2 × 10^6^ cells.

### 2.5. Elastase

Neutrophil degranulation was measured according to the extent of release of the primary granule enzyme, elastase. Neutrophils were incubated at a concentration of 2 × 10^6^ mL^−1^ in HBSS for 10 min at 37°C followed by sequential addition of the test *β*2-agonists (1–1000 nM) and, 20 s later, by fMLP/CB (1 *μ*M : 1 *μ*M), and the tubes then incubated for 10 min at 37°C. The tubes were then transferred to an ice bath, followed by centrifugation at 400 ×g for 5 min to pellet the cells, and the supernatants were decanted and assayed for elastase using a micromodification of a standard colorimetric procedure. Briefly, 125 *μ*L of supernatant was added to the elastase substrate, N-succinyl-L-analyl-L-analyl-L-alanine-*p*-nitroanilide (3 mmol·L^−1^ in DMSO) in 0.05 M Tris-HCl (pH 8.0), and elastase activity was monitored spectrophotometrically at a wavelength of 405 nm. The results are expressed as milliunits enzyme per 2 × 10^6^ cells.

### 2.6. CR3 Expression

Expression of CR3 on resting and fMLP/CB (1 *μ*M : 1 *μ*M)-activated neutrophils in the absence and presence of the test *β*2-agonists (added as above) was measured flow cytometrically. Neutrophils (1 × 10^6^ mL^−1^ final) were incubated in HBSS for 10 min at 37°C followed by addition of the *β*2-agonists (100 nM, fixed, final concentration) and activator. The tubes were incubated for 1 min and the reactions stopped by dilution of the cells in ice-cold medium. Phycoerythrin-labelled monoclonal antibody to CD11b (Beckman Coulter, Miami, FL, USA) was added in a 10 *μ*L volume to 1 mL of cell suspension and CR3 expression analyzed using a Gallios 10C Flow Cytometer (Beckman Coulter). The results were expressed as mean fluorescence intensity.

### 2.7. Measurement of Leukotriene B4 (LTB4) and cAMP

Competitive binding immunoassay procedures (Correlate-EIA; Assay Designs Inc., Ann Arbor, MI, USA) were used to measure LTB4 in the supernatants of fMLP (1 *μ*M) or PAF (200 nM)-activated neutrophils, while cAMP was assayed in the extracts of unstimulated cells in the absence and presence of the test *β*2-agonists. In the case of LTB4, neutrophils (2 × 10^6^ mL^−1^) were preincubated for 10 min at 37°C after which the test *β*2-agonists (10 and 100 nM, final) were added followed 20 s later by fMLP or PAF. Incubation was terminated 5 min later by the addition of an equal volume of ice-cold HBSS to the tubes, which were then held in an ice bath prior to pelleting the cells by centrifugation. The cell-free supernatants were then diluted (1 : 8) and assayed for LTB4, with the results expressed as pg LTB4 per 10^7^ cells.

In the case of cAMP, neutrophils (2 × 10^6^ mL^−1^) were preincubated for 10 min at 37°C after which the test *β*2-agonists (10 and 100 nM) were added and the reactions terminated 20 s later by the addition of an equal volume of ice-cold HBSS to the tubes. Following centrifugation, the supernatants were discarded and cAMP extracted from the cell pellets by addition of 0.1 M HCl for 30 min followed by centrifugation to remove cell debris and the supernatants decanted and assayed for cAMP. These results are expressed as pmol cAMP per 10^7^ cells.

### 2.8. Spectrofluorimetric Measurement of Cytosolic Ca^2+^

Fura-2 acetoxymethyl ester (fura-2AM) was used as the fluorescent, Ca^2+^-sensitive indicator for these experiments [17]. Neutrophils (1 × 10^7^ mL^−1^) were incubated with fura-2AM (2 *μ*M) for 30 min at 37°C in PBS, washed and resuspended in HBSS. The fura-2-loaded cells (2 × 10^6^ mL^−1^) were then preincubated for 10 min at 37°C after which they were transferred to disposable reaction cuvettes which were maintained at 37°C in a Hitachi 650 10 S fluorescence spectrophotometer with excitation and emission wavelengths set at 340 and 500 nm, respectively. After a stable baseline was obtained (±1 min), test agents (1–100 nM) or solvent control were added to the relevant systems followed 20 s later by fMLP (1 *μ*M) or PAF (200 nM) and alterations in fluorescence intensity were monitored over a 5–10 min time course. Alterations in cytosolic Ca^2+^ concentrations following activation of neutrophils with fMLP or PAF were calculated using procedures described in detail elsewhere, either as absolute concentrations [17] or by calculating the areas under the curve using the fura-2/AM tracings [16]. In the case of the latter, the tracings were manually copied, electronically scanned, and saved in jpg format. These images were then imported into the area calculator software program, SketchandCalc, and results are expressed as cm^2^.

A modification of this procedure, namely, Mn^2+^ quenching of fura-2 fluorescence, was used to monitor the effects of the test *β*2-agonists (10 and 100 nM) on store-operated uptake of extracellular Ca^2+^ by activated neutrophils. Briefly, cells (2 × 10^6^ mL^−1^) loaded with fura-2/AM as described above were preincubated for 8 min at 37°C followed by successive addition of Mn^2+^ (600 *μ*M) for a further 2 min period, then the test *β*2-agonists followed 20 s later by fMLP (1 *μ*M) or PAF (200 nM). Ca^2+^ influx was then determined according to the rate and magnitude of quenching of fura-2-fluorescence, using an excitation wavelength of 360 nm, which is an isosbestic wavelength, and an excitation wavelength of 500 nm. These results are presented as the traces from 2 separate experiments, as well as by measurement of the rate of decline in fluorescence intensity at 1 and 4 min following addition of the chemoattractants.

### 2.9. Cell Viability

Neutrophils (1 × 10^6^/mL) were treated with formoterol, indacaterol, or salbutamol, at a final concentration of 100 nM for 10–15 min at 37°C, followed by a 10 min exposure of the cells to propidium iodide (DNA prepstain, Beckman Coulter Miami, FL, USA, 50 *μ*g/mL), at room temperature with flow cytometric detection of uptake of propidium iodide as a marker of cell membrane damage expressed as % viable cells.

### 2.10. Statistical Analysis

With the exception of the results of the oxygen consumption and fura-2/AM fluorescence experiments, some of which are presented as representative traces, the results of each series of experiments are presented as the mean ± SEM values, where *n* equals the number of different donors used in each series of experiments, with the number of replicates for each drug concentration and drug-free control system shown in the figure legends. Levels of statistical significance were determined by comparing the absolute values for each drug-treated system with the corresponding values for the relevant drug-free control systems for each assay using the Mann-Whitney test.

## 3. Results

### 3.1. Production of Reactive Oxygen Species

**Figure 1:**
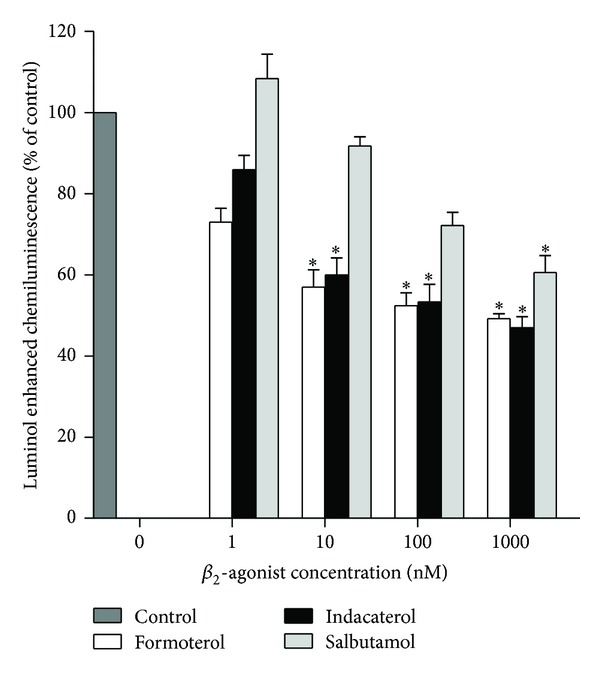
Effects of formoterol, indacaterol, and salbutamol (1–1000 nM) on the luminol-enhanced chemiluminescence responses of neutrophils activated by N-formyl-L-methionyl-L-leucyl-L-phenylalanine (fMLP, 1 *μ*M). The results are expressed as the mean percentage of control ± SEM (*n* = 5) with duplicate values for each drug concentration and control system in each experiment. The absolute values for unstimulated neutrophils and for cells activated with fMLP in the absence of the drugs were 2136 ± 219 and 20394 ± 1447 relative light units respectively; *P < 0.04–0.004 for comparison with the fMLP-activated, drug-free control system.

These results are shown in Figure 1. Treatment of neutrophils with either formoterol or indacaterol at concentrations of 1–1000 nM caused dose-related inhibition of the generation of ROS which achieved statistical significance at concentrations of ≥10 nM (*P* < 0.0188–0.004), thereafter gradually leveling off, possibly due to *β*2AR saturation, with the 2 agents being essentially equipotent. Salbutamol was less effective, only attaining statistically significant inhibition at 1000 nM (*P* < 0.04).

### 3.2. Oxygen Consumption

**Figure 2:**
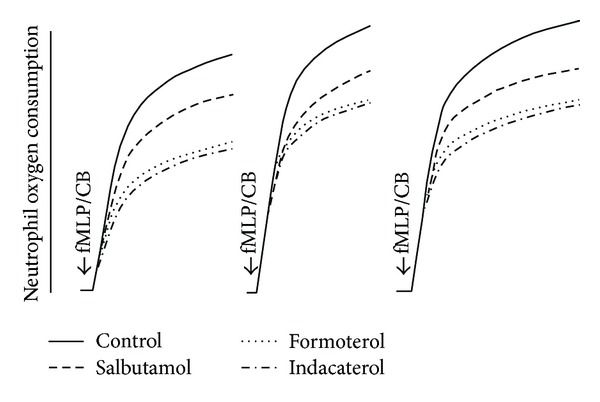
Typical traces from 3 separate experiments (*n* = 3) showing the effects of formoterol, indacaterol, and salbutamol at 100 nM on the magnitude of oxygen consumption by neutrophils activated with N-formyl-L-methionyl-L-leucyl-L-phenylalanine (1 *μ*M)/cytochalasin B (1 *μ*M) (fMLP/CB).

As shown in Figure 2, which depicts the traces from 3 separate experiments using cells from 3 different donors, activation of neutrophils with fMLP/CB was accompanied by a marked, transient increase in O_2_ consumption by the cells which was linear for about 1-2 min, declining rapidly thereafter. Inclusion of the test *β*2-agonists at a fixed final concentration of 100 nM decreased the utilization of O_2_ by these cells. The mean values ± SEMs for the complete series of experiments (*n* = 3) for the fMLP/CB-activated control system and for systems containing formoterol, indacaterol, and salbutamol were 65.4 ± 3, 44.7 ± 3 (*P* < 0.0022 by comparison with the control system), 40.0 ± 4 (*P* < 0.0022), and 50.3 ± 2 (*P* < 0.0022) nmol O_2_ consumed by 2 × 10^6^ cells min^−1^, respectively, over the 1 min period when the response was linear.

### 3.3. Release of Elastase

**Figure 3:**
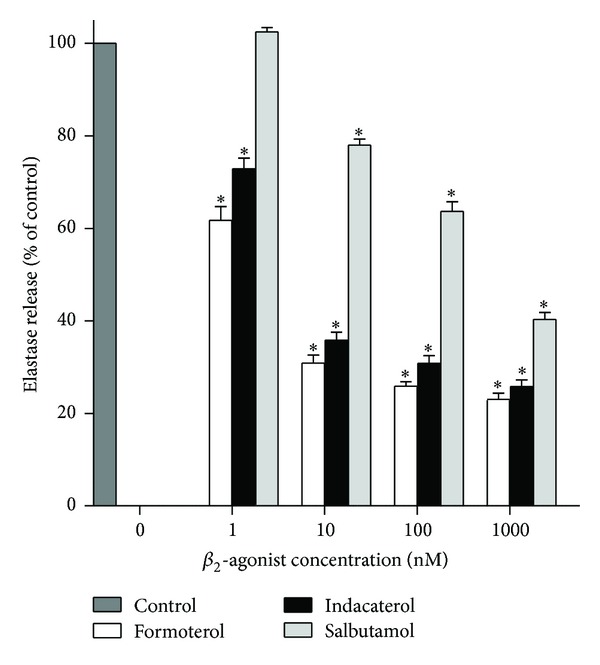
Effects of formoterol, indacaterol, and salbutamol (1–1000 nM) on the release of elastase from neutrophils activated with N-formyl-L-methionyl-L-leucyl-L-phenylalanine (1 *μ*M)/cytochalasin B (1 *μ*M) (fMLP/CB). The results of 3 separate experiments (*n* = 3) with 5 replicates for each drug concentration and control system in each experiment are expressed as the mean percentage of control ± SEM. The absolute values for the unstimulated control system and for cells activated with fMLP/CB were 47 ± 6 and 1551 ± 78 milliunits elastase per 10^7^ cells. *P < 0.0102–0.0001 for comparison with the drug-free control systems.

These results are shown in Figure 3. Treatment of neutrophils with either formoterol or indacaterol at concentrations of ≥ 1 nM caused significant (*P* < 0.0001) dose-related inhibition of the release of elastase, again leveling off at around 10 nM. Salbutamol was less effective, attaining statistically significant inhibition at concentrations of ≥ 10 nM (*P* < 0.0001).

### 3.4. CR3 Expression

Both formoterol and indacaterol, but not salbutamol, at the fixed concentration used (100 nM) caused statistically significant inhibition of expression of CR3 by fMLP/CB-activated neutrophils. The values for the unstimulated control system and for fMLP/CB-activated systems in the absence and presence of formoterol, indacaterol, or salbutamol were 58 ± 4, 121 ± 6, 97 ± 5 (*P* < 0.0045 by comparison with the control system), 99 ± 5 (*P* < 0.0071), and 110 ± 6 (NS) mean fluorescence intensity, respectively, (*n* = 7).

### 3.5. Leukotriene B4

**Figure 4:**
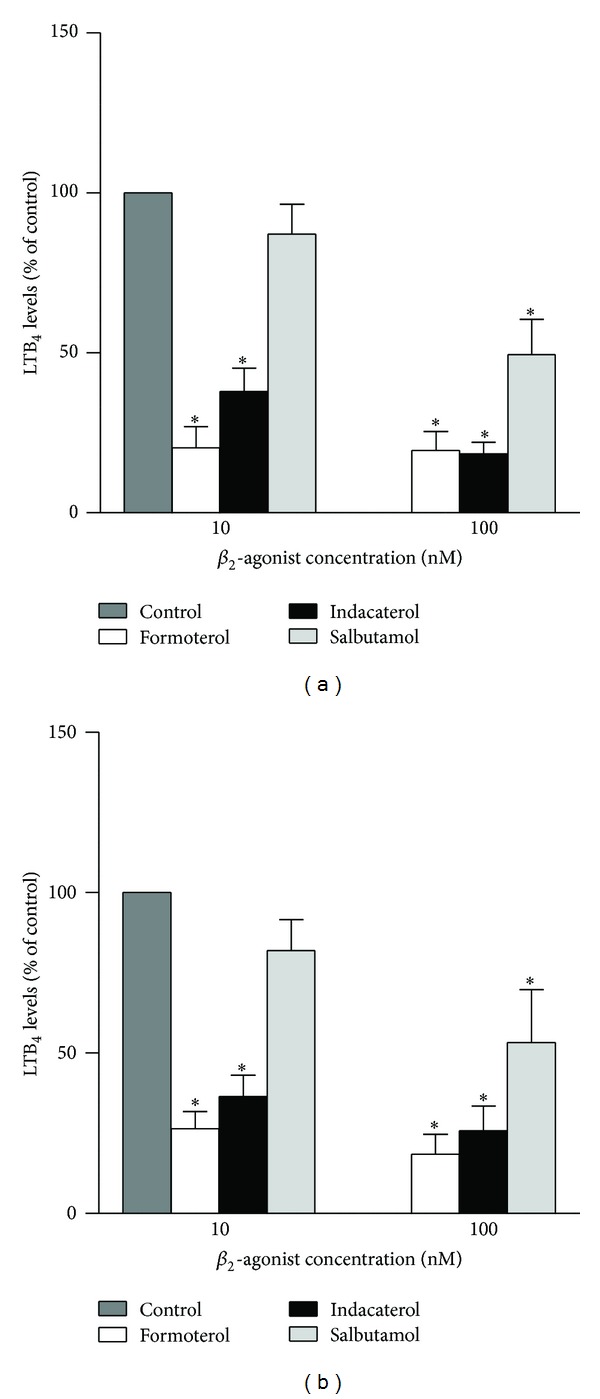
Effects of formoterol, indacaterol, and salbutamol (10 and 100 nM) on the production of LTB_4_ by fMLP- (a) and PAF- (b) activated neutrophils. The results of 5 separate experiments (*n* = 5) with 2 replicates for each drug concentration and control system in each experiment are expressed as the mean percentage of control ± SEM. The absolute values for the unstimulated control system and for cells activated with either fMLP or PAF were 33 ± 7, 1095.4 ± 362.2, and 1546 ± 1108.5 pg mL^−1^. *P < 0.001 for comparison with the drug-free control systems.

The effects of the test *β*2-agonists on the production of LTB4 by neutrophils activated with fMLP or PAF are shown in Figure 4. At the concentrations tested (10 and 100 nM), both formoterol and indacaterol caused dose-related, essentially equivalent, statistically significant (*P* < 0.001) inhibition of LTB4 production activated by either chemoattractant, while the effects of salbutamol were evident at 100 nM only (*P* < 0.001).

### 3.6. Cyclic AMP

**Figure 5:**
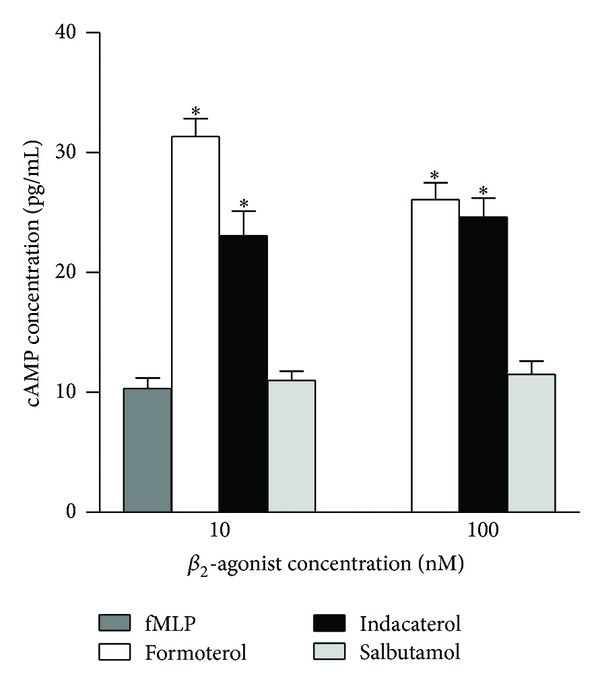
Effects of formoterol, indacaterol, and salbutamol (10 and 100 nM) on cAMP levels in neutrophils. The results of 5 separate experiments (*n* = 5) with 2 replicates for each drug concentration and control system in each experiment are expressed as the mean ± SEM. The absolute value for the control system was 10.4 ± 0.9 pg mL^−1^. *P < 0.001 for comparison with the drug-free control systems.

These results are shown in Figure 5. Treatment of neutrophils with formoterol and indacaterol for 20 s at both concentrations used (10 and 100 nM) resulted in significant (*P* < 0.0001) and comparable elevations in intracellular cAMP, while salbutamol was apparently ineffective at these concentrations. No clear dose-response relationship was evident with formoterol and indacaterol consistent with *β*2AR saturation at 10 nM of these agents.

### 3.7. Cytosolic Ca^2+^

**Figure 6:**
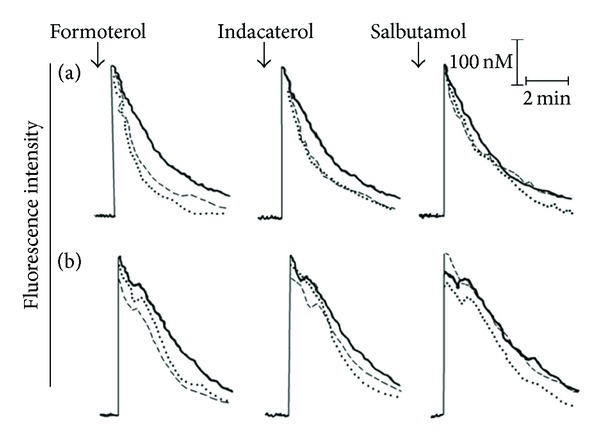
Fura-2 fluorescence traces from 2 representative experiments (*n* = 4–7 in the series) showing the effects of formoterol, indacaterol, and salbutamol (10 and 100 nM) on the alterations in cytosolic Ca^2+^ concentrations following activation of the cells with either 1 *μ*M fMLP (a) or 200 nM PAF (b). The 3 lines in each trace correspond to the control system (—) and systems treated with either 10 nM (_ _) or 100 nM (●●●●●) of each drug; ↓ denotes the addition of either fMLP or PAF.

The results shown in Figure 6 are traces from 2 representative experiments showing the effects of formoterol, indacaterol, and salbutamol (10 and 100 nM) on the alterations in cytosolic Ca^2+^ following activation of the cells with either fMLP or PAF, while data from the full series of experiments are summarized in Table 1. In the case of fMLP, addition of the chemoattractant to neutrophils resulted in the abrupt, characteristic increase in cytosolic Ca^2+^ (fura-2 fluorescence) originating predominantly via mobilization of the cation from intracellular stores, rising from a basal concentration of 89 ± 7 nM to 364 ± 31 nM Ca^2+^. This was followed by a rapid decline over an approximately 2 min period due to clearance of cytosolic Ca^2+^ and a slowing thereafter due to store-operated influx of the cation, respectively [18]. Treatment of the cells with the *β*2-agonists did not affect the magnitude of the initial peak increase in cytosolic Ca^2+^ (not shown). However, as shown in Figure 6 and Table 1, all 3 agents accelerated the rate of decline in fluorescence intensity attaining statistical significance with formoterol and indacaterol at concentrations of 10 nM, but not in the case of salbutamol. These findings are compatible with *β*2-agonist-mediated enhancement of the clearance of Ca^2+^ from the cytosol of fMLP-activated neutrophils.

Activation of neutrophils with PAF also resulted in an abrupt increase in the concentration of cytosolic Ca^2+^ which persisted for about 1 min due to rapid influx of the cation, which, relative to fMLP, was followed by a more gradual clearance of the cation as reported previously [19]. The effects of the 3 *β*2-agonists on PAF-activated alterations in neutrophil cytosolic Ca^2+^ were comparable to those of fMLP-activated cells, namely, no effects on immediate peak responses, followed by accelerated clearance of the cation, with formoterol and indacaterol being most effective (Figure 6, Table 1).

**Figure 7:**
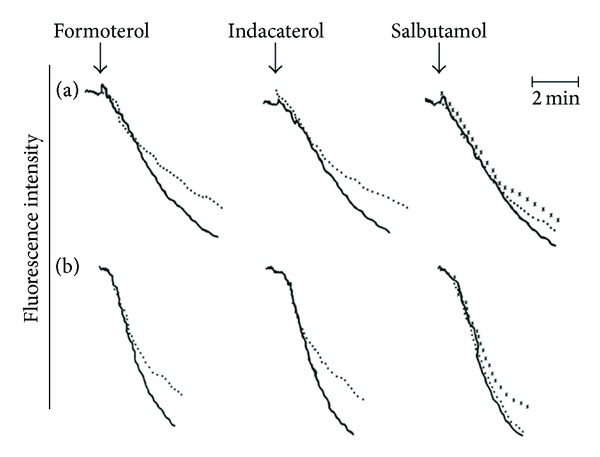
Mn^2+^-quenching of fura-2 fluorescence traces from 2 representative experiments (*n* = 3 in the series) showing the effects of formoterol, indacaterol (100 nM), and salbutamol (100 and 1000 nM) on the alterations in cytosolic Ca^2+^ concentrations following activation of the cells with either 1 *μ*M fMLP (a) or 200 nM PAF (b). The 3 lines in each trace correspond to the control system (—) and systems treated with 100 nM of each drug (●●●●●) or 1000 nM salbutamol (xxxx); ↓ denotes the addition of either fMLP or PAF.

The effects of the 3 *β*2-agonists on store-operated influx of extracellular Ca^2+^ following activation of neutrophils with either fMLP or PAF are shown in Figure 7 which are the traces from 2 representative experiments; these are summarized in Table 2. At the concentrations tested (10 and 100 nM), formoterol and indacaterol effectively inhibited the influx of Ca^2+^ by cells activated with either fMLP or PAF, while the effects of salbutamol were evident at a concentration of 1000 nM.

### 3.8. Cell Viability

Exposure of the neutrophils to formoterol, indacaterol, or salbutamol at a final concentration of 100 nM had no effect on cell viability according to propidium iodide exclusion, the respective values being 95.9 ± 0.5%, 95.9 ± 0.5% and 96.0 ± 0.5% viability relative to a control value of 96.4 ± 0.6% (data from eight determinations).

## 4. Discussion

The findings of the current study demonstrate that formoterol and indacaterol effectively suppress a spectrum of proinflammatory activities of activated human neutrophils* in vitro*, while salbutamol is much less effective. The different magnitudes of anti-inflammatory activity observed with formoterol, indacaterol, and salbutamol are most likely due to variability in the intrinsic efficacy of these agents. Formoterol, a full agonist [5] and indacaterol, a near full agonist [20], have higher intrinsic efficacy than the partial agonist, salbutamol [5]. With respect to formoterol and indacaterol, all of the neutrophil functions tested were suppressed at low nanomolar (1–10 nM) concentrations of these agents including the generation of ROS and LTB4, release of the primary granule protease, elastase, and expression of the *β*2-integrin, CR3. These neutrophil-derived mediators of inflammation are intimately involved in the immunopathogenesis not only of COPD, but also other acute and chronic inflammatory diseases of the airways such as severe persistent bronchial asthma, COPD, cystic fibrosis, bronchiolitis obliterans, and acute respiratory distress syndrome, resulting in pulmonary abnormalities including bronchial hyperresponsiveness and obstruction, mucus hypersecretion, and airway remodeling [10, 21–27].

From a mechanistic perspective, exposure of neutrophils to formoterol and indacaterol, at concentrations equivalent to those which suppressed neutrophil reactivity, resulted in *β*2AR-mediated increases in intracellular cAMP. This, in turn, apparently underpins the increased efficacy of restoration of Ca^2+^ homeostasis to neutrophils activated with either fMLP or PAF, with consequent attenuation of Ca^2+^-dependent proinflammatory activity. Several mechanisms have been described, all involving cAMP-dependent protein kinase (PKA), whereby cAMP promotes accelerated clearance of Ca^2+^ from the cytosol of activated neutrophils and other cell types (reviewed in 5). However, PKA-mediated enhancement of the efficacy of the Ca^2+^ resequestering endomembrane Ca^2+^-ATPase, which diverts mobilized cytosolic Ca^2+^ back into storage compartments [28], is likely to be the predominant mechanism operative in the setting of the observed *β*2-agonist/neutrophil interactions. This contention is based on the following observations: (i) the lack of effect of the test agents on the immediate peak cytosolic Ca^2+^ response following exposure of the cells to fMLP or PAF, excluding interference with phospholipase C and generation of Ca^2+^-mobilizing inositol triphosphate, and (ii) decreased store-operated influx of extracellular Ca^2+^ which is probably secondary to efficient utilization of cytosolic Ca^2+^ for store refilling [28].

The apparent disconnect between the failure to detect elevated levels of intracellular cAMP in the setting of moderate inhibition of oxygen consumption, generation of ROS and LTB4, and release of elastase following treatment of neutrophils with salbutamol at a concentration of 100 nM may be attributed to several reasons. Firstly, as a consequence of both the partial agonist activity and comparatively low partition coefficient of salbutamol, sustained elevations in cAMP may be difficult to detect due to rapid hydrolysis of the cyclic nucleotide by the cyclic AMP-specific phosphodiesterase, PDE4. This contention is substantiated by the findings of a previous study demonstrating that the elevating actions of salbutamol on neutrophil cAMP are dependent on the inclusion of an inhibitor, either selective or nonselective, of PDEs [16]. Alternatively, salbutamol may suppress the proinflammatory activities of neutrophils by mechanisms unrelated to *β*2AR-mediated activation of adenylyl cyclase [29]. Importantly, the anti-inflammatory effects of formoterol and indacaterol could not be attributed to cellular cytotoxicity.

Despite a considerable body of evidence demonstrating potent, cAMP-dependent, suppressive effects of *β*2-agonists on cells of the innate and adaptive immune systems* in vitro* [3, 5, 28], as well as anti-inflammatory activity in animal models of experimental airways inflammation [30], compelling evidence for the existence of these activities in the clinical setting has not been forthcoming [6, 7]. This may be attributable to several possible causes. These are (i) aspects of molecular structure, as mentioned above, such as those in the case of formoterol and indacaterol which confer full agonist activity, good partition coefficients, and long duration of action, as opposed to salbutamol [5]; (ii) failure to attain adequate concentrations in the airways, although inhalation of 24 *μ*g formoterol has been estimated to result in a local concentration of 11 nM [16], which would be effective for this agent, but ineffective in the case of salbutamol; and (iii) lower numbers of *β*2ARs on inflammatory cells relative to airway smooth muscle cells [8, 11], possibly resulting in an increased propensity to undergo receptor desensitization [8]. Of these, *β*2AR desensitization is likely to be the major obstacle to harnessing the anti-inflammatory potential of formoterol and indacaterol in the clinical setting. In addition to augmenting *β*2AR expression by combining *β*-agonists with inhaled corticosteroids [31, 32], potential strategies to overcome receptor desensitization in the clinical setting include adjunctive therapy using selective and nonselective inhibitors of cyclic AMP PDEs and possibly anticholinergics such as the long-acting antimuscarinic, tiotropium [5].

Numerous clinical trials have confirmed the clinical efficacy of indacaterol for the treatment of patients with moderate and severe COPD [33]. In this clinical setting indacaterol provides extended relief of airflow obstruction and dyspnoea and reduces the frequency of exacerbations, resulting in symptomatic relief and a decreased requirement for rescue medications, thereby improving patient quality of life and adherence to therapy [10, 33, 34]. Indacaterol also appears to have a favourable safety profile. In the case of the cardiovascular system for example, 14 days of therapy did not induce any prolongation of the QT interval [35]. This is important as many patients with COPD are elderly and may have associated comorbid cardiovascular disorders. A mild and transient cough is a common adverse effect described by patients treated with indacaterol [34].

In conclusion, indacaterol, like formoterol, effectively targets the potentially harmful activities of stimulated neutrophils* in vitro*. These anti-inflammatory, albeit secondary, actions of both agents may contribute to their therapeutic efficacy in COPD.

## Figures and Tables

**Table 1:** Effects of formoterol, indacaterol, and salbutamol (1, 10, 100, and 1000 nM) on the cytosolic Ca^2+^ concentrations (area under curve) of FMLP- and PAF-activated neutrophils.

Agent	FMLP Mean ± SEM (cm^2^)	PAF Mean ± SEM (cm^2^)
Control (DMSO)	8.6 ± 0.64	13.8 ± 0.53
Formoterol 1 nM	ND	10.9 ± 0.90
Indacaterol 1 nM	ND	12.2 ± 1.34
Salbutamol 1 nM	ND	14.3 ± 1.20
Formoterol 10 nM	4.8 ± 0.64*	10.9 ± 0.74*
Indacaterol 10 nM	5.6 ± 0.56*	10.9 ± 1.08*
Salbutamol 10 nM	7.3 ± 0.50	13.6 ± 1.25
Formoterol 100 nM	4.5 ± 0.28*	9.8 ± 0.90*
Indacaterol 100 nM	5.1 ± 0.57*	10.7 ± 0.90*
Salbutamol 100 nM	7.7 ± 0.74	12.8 ± 1.40
Formoterol 1000 nM	ND	8.0 ± 0.74*
Indacaterol 1000 nM	ND	8.4 ± 0.90*
Salbutamol 1000 nM	ND	10.3 ± 1.90

ND: not done.

*P = 0.02–0.0003.

**Table 2:** Effects of formoterol, indacaterol, and salbutamol on Mn^2+^ quenching of fura-2 fluorescence in FMLP- and PAF-activated neutrophils.

Agent	FMLP-activated neutrophils		PAF-activated neutrophils	
	Magnitude of decrement at 1 minute (cm) Mean ± SEM	Magnitude of decrement at 4 minutes (cm) Mean ± SEM	Magnitude of decrement at 1 minute (cm) Mean ± SEM	Magnitude of decrement at 4 minutes (cm) Mean ± SEM
Control (DMSO)	1.40 ± 0.09	5.40 ± 0.30	2.7 ± 0.15	7.4 ± 0.67
Formoterol 100 nM	1.24 ± 0.09	3.70 ± 0.13*	2.5 ± 0.16	5.1 ± 0.55*
Indacaterol 100 nM	1.30 ± 0.07	3.74 ± 0.19*	2.4 ± 0.17	5.2 ± 0.50
Salbutamol 100 nM	1.60 ± 0.09	5.30 ± 0.22	2.7 ± 0.17	6.8 ± 0.73

*P = 0.04–0.009.
